# Resting‐state functional brain networks in adults with a new diagnosis of focal epilepsy

**DOI:** 10.1002/brb3.1168

**Published:** 2018-11-28

**Authors:** Batil K. Alonazi, Simon S. Keller, Nicholas Fallon, Valerie Adams, Kumar Das, Anthony G. Marson, Vanessa Sluming

**Affiliations:** ^1^ Department of Psychological Sciences, Institute of Psychology, Health and Society University of Liverpool Liverpool UK; ^2^ Department of Radiology and Medical Imaging Prince Sattam Bin Abdulaziz University Al Kharj Saudi Arabia; ^3^ Department of Molecular and Clinical Pharmacology, Institute of Translational Medicine University of Liverpool Liverpool UK; ^4^ The Department of Neuroradiology The Walton Centre NHS Foundation Trust Liverpool UK; ^5^ Liverpool Magnetic Resonance Imaging Centre (LiMRIC) University of Liverpool Liverpool UK

**Keywords:** brain connectivity, cognitive dysfunction, new‐onset seizures, treatment outcome

## Abstract

**Objectives:**

Newly diagnosed focal epilepsy (NDfE) is rarely studied, particularly using advanced neuroimaging techniques. Many patients with NDfE experience cognitive impairments, particularly with respect to memory, sustained attention, mental flexibility, and executive functioning. Cognitive impairments have been related to alterations in resting‐state functional brain networks in patients with neurological disorders. In the present study, we investigated whether patients with NDfE had altered connectivity in large‐scale functional networks using resting‐state functional MRI.

**Methods:**

We recruited 27 adults with NDfE and 36 age‐ and sex‐matched healthy controls. Resting‐state functional MRI was analyzed using the Functional Connectivity Toolbox (CONN). We investigate reproducibly determined large‐scale functional networks, including the default mode, salience, fronto‐parietal attention, sensorimotor, and language networks using a seed‐based approach. Network comparisons between patients and controls were thresholded using a FDR cluster‐level correction approach.

**Results:**

We found no significant differences in functional connectivity between seeds within the default mode, salience, sensorimotor, and language networks and other regions of the brain between patients and controls. However, patients with NDfE had significantly reduced connectivity between intraparietal seeds within the fronto‐parietal attention network and predominantly frontal and temporal cortical regions relative to controls; this finding was demonstrated including and excluding the patients with brain lesions. No common alteration in brain structure was observed in patients using voxel‐based morphometry. Findings were not influenced by treatment outcome at 1 year.

**Conclusions:**

Patients with focal epilepsy have brain functional connectivity alterations at diagnosis. Functional brain abnormalities are not necessarily a consequence of the chronicity of epilepsy and are present when seizures first emerge.

## 1 INTRODUCTION

Neuroimaging approaches have provided important insights into long‐standing, typically treatment‐refractory epilepsy. Sophisticated MRI approaches in particular have provided a deeper understanding of the biological mechanisms underlying the development of focal and generalized epilepsies (Bernhardt, Hong, Bernasconi, & Bernasconi, 2013; Duncan, 2005; Koepp & Woermann, 2005), and have recently been used to gain insights into response to surgical intervention in patients with refractory focal epilepsy (Bonilha et al., 2015; Keller et al., 2017, 2015; Munsell et al., 2015). Comparatively, newly diagnosed epilepsy is rarely studied despite this being a key point in time to understand the underlying biology of epilepsy and to identify potential interventions and biomarkers for seizure and cognitive outcomes. The translation of what we understand in long‐standing epilepsy to people with a new diagnosis of epilepsy is confounded by several factors, including the chronic effects of seizures and anti‐epileptic drugs (Pohlmann‐Eden, Crocker, & Schmidt, 2013). This lack of investigation is most notably due to access to patients; many specialist and academic centres do not see epilepsy until it is well established. As such, advanced imaging studies—which yield important structural and functional information beyond what can be obtained from conventional neuroimaging in the context of standard clinical care—have not been published in patients with newly diagnosed epilepsy (Pohlmann‐Eden, 2011; Pohlmann‐Eden et al., 2013).

Focal onset epilepsy is more prevalent than idiopathic generalized epilepsy (IGE; Sander & Shorvon, 1996) and is more commonly associated with pharmacoresistance (Kwan & Brodie, 2000) and memory dysfunction (van Rijckevorsel, 2006). There are only few studies of adults with newly diagnosed focal epilepsy (NDfE) using MRI approaches, most of which have used conventional applications (i.e., volumetric image analysis techniques applied to clinically acquired T1‐weighted images). Studies have revealed that 65%–96% adults with NDfE have no MRI observed lesion (Liu et al., 2002; Van Paesschen, Duncan, Stevens, & Connelly, 1997, 1998). Most volumetric MRI studies of the hippocampus reveal no significant difference between in patients with NDfE and healthy controls (Liu et al., 2001, 2002; Salmenpera et al., 2005). One study revealed mild hippocampal changes at diagnosis, which contrasted to substantial hippocampal atrophy in patients with chronic focal epilepsy (Saukkonen et al., 1994). In one longitudinal study of adults with newly diagnosed focal temporal lobe epilepsy, 24/24 were MRI‐negative at baseline, whereas a single patient developed hippocampal sclerosis in a follow‐up scan approximately 3 years later (Briellmann, Berkovic, Syngeniotis, King, & Jackson, 2002). Cerebellar volume is normal at diagnosis of focal epilepsy (Hagemann et al., 2002). Generally speaking, there have been no reports of common gross brain structural changes in adults with NDfE when assessed using volumetric MRI approaches. There is a need to understand changes in brain structure and function using advanced neuroimaging techniques at the earliest reliable time point following a diagnosis of human epilepsy.

Adults with epilepsy may be cognitively impaired at the time of diagnosis. Drug‐naïve patients with NDfE show significant impairments in memory, sustained attention, executive functioning, mental flexibility, and psychomotor speed relative to healthy volunteers (Aikia, Kalviainen, & Riekkinen, 1995; Aikia, Salmenpera, Partanen, & Kalviainen, 2001; Kalviainen, Aikia, Helkala, Mervaala, & Riekkinen, 1992; Prevey, Delaney, Cramer, & Mattson, 1998; Pulliainen Kuikka, & Jokelainen, 2000; Taylor et al., 2010). One 12‐month follow‐up study revealed that performance on some of these cognitive domains further deteriorated (Baker, Taylor, & Aldenkamp, 2011); another study reported no subsequent significant worsening of verbal memory performance in patients impaired at diagnosis and that memory dysfunction was not related to hippocampal volume (Aikia et al., 2001). Cognitive deficits—which along with spontaneous seizures contribute to impaired quality of life in epilepsy (Engelberts et al., 2002)—are therefore not necessarily a result of the chronicity of the disorder, including the recurrent seizures and chronic use of anti‐epileptic drugs, and are therefore likely to be the result of epileptogenesis. There are, however, no existing neuroimaging insights of the underlying aetiology and mechanisms of cognitive dysfunction in NDfE.

Functionally connected large‐scale networks that have significance for particular cognitive domains can be delineated in the human brain using resting‐state functional MRI. These neuroimaging approaches have provided significant insights into cognitive dysfunction in people with neurological, neurodegenerative, and neuropsychiatric disorders (Cataldi, Avoli, & Villers‐Sidani, 2013; Li et al., 2015; Woodward & Cascio, 2015). Three of the most investigated networks include the default mode network (key role in internally directed or self‐generated thought; Andrews‐Hanna, Smallwood, & Spreng, 2014; Greicius, Krasnow, Reiss, & Menon, 2003; Raichle et al., 2001) has dynamic roles in cognitive processing (Ichesco et al., 2012) and is compromised in patients with loss of consciousness (Vanhaudenhuyse et al., 2010), the salience network (key roles in communication, social behaviour, self‐awareness, and multiple facets of cognition; Menon, 2015), and the fronto‐parietal attention network (key roles in attention, cognitive control, and executive functioning; Markett et al., 2014; Schmidt, Burge, Visscher, & Ross, 2016). Alterations in these three functional networks have been reported in patients with chronic temporal lobe epilepsy and idiopathic generalized epilepsy (de Campos, Coan, Lin Yasuda, Casseb, & Cendes, 2016; Kay et al., 2013; Wei et al., 2015), and such alterations have been inferred to underlie cognitive impairment in these patient groups. Given that the neuropsychological literature suggests that patients with NDfE have particular cognitive impairments in the attention, cognitive control, and executive function domains, there may be alterations in functional connectivity within the fronto‐parietal attentional network, or between nodes in this network and other brain regions. However, there are no published studies that have investigated functional networks in patients with NDfE.

There were two primary objectives of the present study. We sought to determine whether core functional networks are altered in patients with a new diagnosis of focal epilepsy relative to a cohort of healthy controls using resting‐state functional MRI. We hypothesized abnormalities of functional networks that are known to play a role in the facets of cognition function previously demonstrated to be impaired in patients with NDfE (particularly memory, attention, and executive function), most notably, the fronto‐parietal attention network. Secondly, in order to determine whether functional network alterations existed in patients in the absence of gross structural abnormalities, we performed voxel‐based morphometry (VBM) comparisons of regional grey matter volume between patients and controls (Keller & Roberts, 2008; Keller et al., 2015).

## 2 METHODS

### 2.1 Patients

We recruited 27 patients with NDfE (mean age, 33.1 years [*SD* 11.3], range 18–57; 12 [44%] females) attending outpatient clinics at the Walton Centre NHS Foundation Trust in Liverpool. Focal epilepsy was diagnosed by expert epileptologists based on the latest International League Against Epilepsy (ILAE) operational classifications (Fisher et al., 2017). Diagnostic features consistent with focal epilepsy were based on detailed assessment of seizure semiology. Demographic and clinical information for patients is provided in Table 1. In order to increase the number of patients recruited into this study, we did not constrain recruitment to drug‐naïve patients. We scanned patients an average of 3.7 months after diagnosis (*SD* 2.9, range 1–11 months). We did not anticipate any deleterious effects on brain function or cognition within this time period. Exclusion criteria included provoked seizures (e.g., drug induced), acute symptomatic seizures (e.g., acute brain haemorrhage or brain injury), primary generalized seizures, unclassified seizures, and known progressive neurological disease (e.g., brain tumour, Alzheimer’s disease). All patients underwent EEG as part of their clinical investigations using the conventional 10–20 system. All patients were followed up 1 year after functional MRI to determine response to AED therapy. We also studied 36 age‐matched neurologically and neuropsychiatrically healthy volunteers (mean age 33.7 years [*SD* 11.6], range 18–58; 22 [61%] females).

**Table 1:** Patient clinical data. Age is years. Time between diagnosis and resting‐state functional MRI (Dx > fMRI) is months

	Age	Sex	EEG	MRI Report	Medication	Dx > fMRI	Seizures between Dx & MRI	Neurological History	Treatment outcome
1	18	M	N	FCD & Hipp R < L	LMT 400 mg	6	Multiple FSIA	No neurological history	PS
2	37	F	N	Normal	LMT 1,000 mg	2	FTBTC	Syncope followed by concussive seizure	SF
3	39	M	N	Frontal focal gliosis	LMT 100 mg	7	No Seizures	2 FTBTC & brain injury age of 15	SF
4	57	M	N	FCD	LEV 1,000 mg	8	FSIA	FTBTC & pituitary cyst	SF
5	43	F	N	Normal	LEV 1,000 mgs	1	FSIA	Headaches & previous seizures	SF
6	30	M	N	Normal	LAM 150 mg	7	Single FSIA	No neurological history	SF
7	28	F	N	Normal	LEV 1,000 mg	5	No Seizures	FSIA & FTBTC	PS
8	37	M	A	Normal	ZNS 200 mg	8	2 FSIA	FSIA & FTBTC	PS
9	30	M	N	Hippo L < R	LMT 500 mg	8	FSIA	Von Willebrand disease	PS
10	22	M	N	Normal	ZNS 150 mg	1	No Seizures	FTBTC	SF
11	37	M	N	Normal	LMT 150 mg	2	No Seizures	History of FC	SF
12	38	F	N	Multiple WM hypointensity; haemosiderin and suggestive of previous microhaemorrhages	ZNS 250 mg	5	FSIA & FTBTC	Previous hypoxic brain injury	SF
13	37	F	N	Normal	ZNS 500 mg	1	No Seizures	FSA	SF
14	18	F	N	Normal	LMT 150 mg	11	4 FSIA & FTBTC	No neurological history	PS
15	54	F	N	Normal	LMT 100 mg	1	6 FSIA	FTBTC and history of FC	SF
16	41	F	A	Normal	LEV 500 mg	5	FSIA & FTBTC	FTBTC	SF
17	25	F	N	Normal	LMT 200 mg	3	FSIA	No neurological history	SF
18	18	M	A	Normal	LMT 50 mg	2	FSIA	FTBTC	PS
19	56	M	N	Normal	LMT 150 mg	1	No Seizures	FSA & FTBTC	PS
20	41	F	N	Normal	LMT 300 mg	2	No Seizures	FSIA & FTBTC	SF
21	22	M	N	R hippo change^a^	LMT 50 mg	3	No Seizures	FTBTC	SF
22	23	M	N	Normal	LMT 150 mg	3	No Seizures	FTBTC	SF
23	20	F	N	Normal	LMT 100 mg	1	No Seizures	No neurological history	PS
24	32	M	N	Right FL gliosis, encephalomalacia & CC atrophy; left posterior gliosis	LEV 1,000 mg	1	No Seizures	FTBS & previous brain injury	PS
25	38	F	N	Normal	LEV 1,000 mg	2	No Seizures	FTBTC	PS
26	28	M	N	Normal	LMT 150 mg	1	No Seizures	2 FTBTC	SF
27	24	M	N	Normal	Unknown	2	No Seizures	No neurological history	SF

A, abnormal; CPS, complex partial seizure; F, female; FC, febrile convulsions; FCD, focal cortical dysplasia; FSA, focal seizure, aware (formerly simple partial seizure (Fisher et al., 2017)); FSAI, focal seizure awareness impaired (formerly complex partial seizure (Fisher et al., 2017)); FTBTC, focal to bilateral tonic–clonic (formerly generalized tonic–clonic seizure (Fisher et al., 2017)); Hippo, hippocampal volume; L, left; LEV, Levetiracetam; LMT, Lamotrigine; M, male; N, normal; PS, persistent seizures; R, right; SF, seizure free; WM, white matter; ZNS, Zonisamide.

a Right hippocampal change was observed on Fluid‐Attenuated Inversion Recovery (FLAIR) MRI only.

### 2.2 MRI acquisition

All patients and controls were scanned at the Liverpool Magnetic Resonance Imaging Centre (LiMRIC) at the University of Liverpool, and we acquired 3D T1‐weighted and resting‐state functional MRI data using a 3 T MR system (Siemens Trio). For the T1‐weighted data, we acquired Magnetization Prepared Rapid Gradient Echo (MPRAGE) sequence with the following parameters: TE = 5.57 ms; TR = 2040 ms; TI = 1,100 ms; slice thickness = 1 mm; voxel size = 1 mm × 1 mm; 176 slices; flip angle = 8. The resting‐state functional MRI data were acquired using a 6‐min T2‐weighted sequence and the following parameters: TE = 30 ms; TR = 2,000 ms; slice thickness = 3.5 mm; voxel size = 3 mm × 3 mm; 180 volumes; 32 slices; flip angle = 90. For the resting‐state functional MRI, participants were asked to remain awake with their eyes closed. We additionally acquired isotropic 3D T2‐weighted (turbo spin echo with variable flip angle; TE = 355 ms; TR = 3,000 ms; slice thickness = 1 mm; voxel size = 1 mm x 1 mm; Turbo factor = 209) and T2‐Fluid‐Attenuated Inversion Recovery (TE = 353 ms; TR = 6,000 ms; slice thickness = 1 mm; voxel size = 1 mm x 1 mm; Turbo factor = 221) images for diagnostic appraisal and reporting of incidental findings in all subjects.

### 2.3 Resting‐state functional analysis

Resting‐state functional data were spatially preprocessed using SPM12 (Welcome Trust Centre for Neuroimaging, University College London, United Kingdom; http://www.fil.ion.ucl.ac.uk/spm/) running in Matlab v.9.0 (The Mathworks Inc, USA). Functional data were realigned, slice‐time corrected, spatially normalized to the Montreal Neurological Institute (MNI) space using the normalized EPI template image in SPM, and spatially smoothed with an 8‐mm full‐width half‐maximum Gaussian kernel. Motion parameters from realignment were evaluated, and a motion artefact threshold (translation >3 mm, rotation >1°) was employed for exclusion (Fallon, Chiu, Nurmikko, & Stancak, 2016). No participants displayed gross movements to require exclusion. For subsequent analyses, each participant’s T1‐weighted MPRAGE image was automatically segmented into grey matter, white matter, and cerebrospinal fluid and normalized to MNI space using the Computational Anatomy Toolbox (CAT12; http://www.neuro.uni-jena.de/cat/) running in SPM12 (see VBM methods).

**Figure 1:**
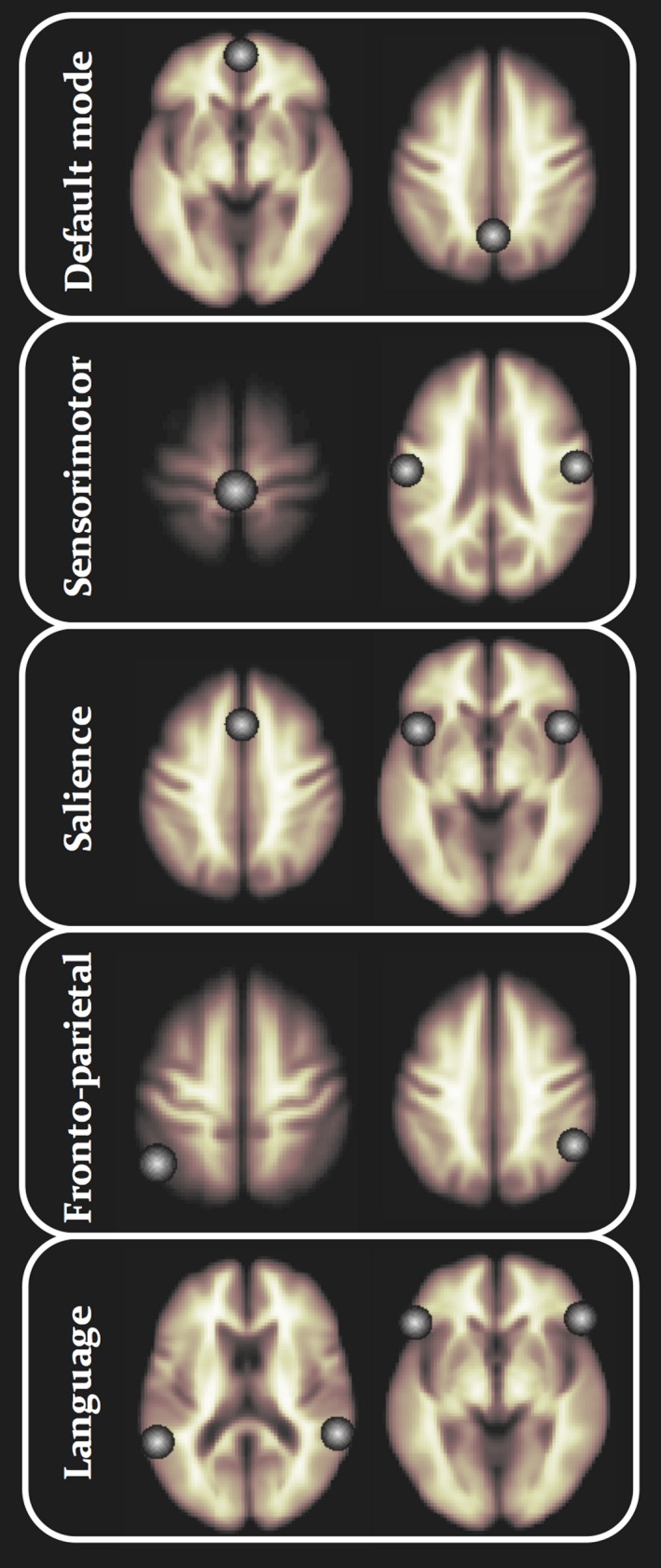
Location of seeds for each resting‐state network. See Table 2 for anatomical locations and coordinates

Spatially preprocessed resting‐state functional data were analyzed using the Functional Connectivity Toolbox (CONN) (Whitfield‐Gabrieli & Nieto‐Castanon, 2012) running in Matlab. CONN implements a component‐based noise correction method (Behzadi, Restom, Liau, & Liu, 2007) to reduce physiological and extraneous noise, providing interpretative information on correlated and anticorrelated functional brain networks. Blood‐oxygen‐level‐dependent (BOLD) signal from the cerebral white matter and ventricles was removed prior to seed‐based connectivity analysis using principal component analysis of the multivariate BOLD signal within each these masks obtained from the segmented T1‐weighted MPRAGE scans (Fallon et al., 2016; Woodward, Rogers, & Heckers, 2011). BOLD data were bandpass filtered (0.008–0.09 Hz) to reduce low‐frequency drift and noise effects. We generated seed‐to‐voxel connectivity maps for each individual for the following reproducibly demonstrated functional networks: the default mode, salience, fronto‐parietal attention, language, and sensorimotor networks. These networks were chosen as they have been intimately associated with aspects of cognitive functioning disrupted in NDfE (Aikia et al., 1995, 2001; Ichesco et al., 2012; Kalviainen et al., 1992; Markett et al., 2014; Menon, 2015; Prevey et al., 1998; Pulliainen et al., 2000; Schmidt et al., 2016; Taylor et al., 2010) and/or have been demonstrated to be significantly altered in refractory epilepsy (de Campos et al., 2016; Kay et al., 2013; Wei et al., 2015). Seeds were 10‐mm‐diameter spheres; the spatial coordinates and anatomical location of network seeds are provided in Table 2 and illustrated in Figure 1. These seeds are provided in the CONN software, and represent core and reproducibly demonstrated topological nodes within each resting‐state network. The reasoning for identification and use of these seeds is described in greater detail by the originators of CONN (Whitfield‐Gabrieli et al., 2011). We investigated functional networks generated from individual seeds separately (i.e., not averaged over seed regions within a given network); this resulted in 14 analyses (two seeds each for default mode and fronto‐parietal networks, three seeds each for sensorimotor and salience networks, and four seeds for the language network; Table 2).

**Table 2:** Seed regions used to generate resting‐state networks. See Figure 1 for visualization of anatomical location of seeds

Network	Anatomical region	BA	*x*	*y*	*z*
Default mode	Medial prefrontal cortex	10	1	55	−3
	Posterior parietal cortex	7	1	−61	38
Sensorimotor	Primary motor area	4	0	−31	67
	Precentral gyrus, left	6	−55	−12	29
	Precentral gyrus, right	6	56	−10	29
Salience	Anterior cingulate gyrus	32	0	22	35
	Anterior insula, left	13	−44	13	1
	Anterior insula, right	13	47	14	0
Fronto‐parietal	Intraparietal sulcus, left	39	−46	−58	49
	Intraparietal sulcus, right	39	52	−52	45
Language	Posterior superior temporal gyrus, left	22	−57	−47	15
	Posterior superior temporal gyrus, right	22	59	−42	13
	Inferior frontal gyrus (pars triangularis), left	45	−51	26	2
	Inferior frontal gyrus (pars triangularis), right	45	54	28	1

Individual correlation maps were generated in the CONN toolbox by extracting the mean resting‐state BOLD time course from each seed ROI and calculating correlation coefficients with the BOLD timecourse of each voxel throughout the whole brain. The resulting coefficients were converted to normally distributed scores using Fisher's transformation to give maps of voxel‐wise functional connectivity for each seed ROI for each subject. The value of each voxel throughout the whole brain represents the relative degree of functional connectivity with each seed (Whitfield‐Gabrieli et al., 2011). These maps were subsequently used for second‐level analysis of relative functional connectivity using a two‐sided independent *t* test, implemented in the CONN toolbox, to investigate differences in seed‐to‐voxel connectivity between groups.

Participant motion parameters were included as within‐subject first‐level covariates. To determine between‐subject effects in resting‐state functional networks, group (patients and controls), presence of MRI lesion, patient seizure outcome status at 1‐year follow‐up, age and gender were included as second‐level covariates. As in previous studies (Fallon et al., 2016; Ichesco et al., 2012; Woodward et al., 2011), we performed voxel‐wise statistical analysis over the entire brain using an uncorrected level (*p* < 0.001) before a false discovery rate (FDR) correction was applied at the cluster level (*p* < 0.05).

### 2.4 Voxel‐based morphometry

VBM was performed using a similar approach as previously described (Keller et al., 2015) but using CAT12 running in SPM12 (as opposed to the VBM8 toolbox running in SPM8). CAT12 includes improvements to the image preprocessing pipeline and has been suggested to provide an improved method for the identification of brain structural abnormalities in patients with epilepsy over previous VBM applications (Farokhian, Beheshti, Sone, & Matsuda, 2017). Briefly, the T1‐weighted MPRAGE images were automatically segmented into grey matter, white matter, and cerebrospinal fluid tissue classes, and spatially normalized to MNI space using DARTEL (Ashburner, 2007). Default options were chosen in the CAT12 batch editor (http://dbm.neuro.uni-jena.de/cat12/CAT12-Manual.pdf). Grey matter and white matter normalized images were smoothed with an isotropic Gaussian kernel of 8 mm. Grey matter and white matter comparisons were made between groups on a voxel‐by‐voxel basis using a full factorial model, including age and sex as confounding covariates. Groups included controls, patients with gross lesions, and patients with no lesion. Only results surviving multiple whole‐brain corrections using the familywise error (FWE) rate (*p* < 0.05) are reported, based on previous recommendations (Keller & Roberts, 2008).

## 3 RESULTS

### 3.1 Patient clinical data

**Figure 2:**
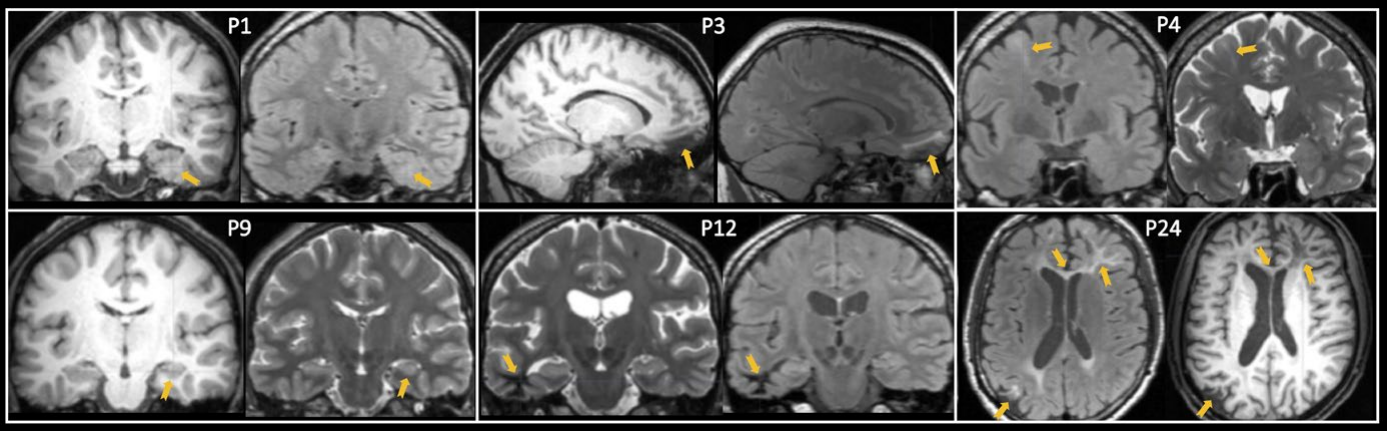
Lesions identified in the present study (see Table 1 for corresponding information). Patient 1 (P1): mesial temporal focal cortical dysplasia and atrophy of ipsilateral hippocampal head on T1‐weighted (left) and T2‐FLAIR (right) images; P3: orbitofrontal gliosis on T1‐weighted (left) and T2‐FLAIR (right) images; P4: focal cortical dysplasia of middle frontal gyrus on T2‐FLAIR (left) and T2‐weighted (right) images; P9: unilateral hippocampal atrophy on T1‐weighted (left) and T2‐weighted (right) images; P12: temporal lobe white matter alteration on T2‐weighted (left) and T2‐FLAIR (right) images; P24: frontal lobe gliosis and encephalomalacia, corpus callosum atrophy and contrecoup posterior gliosis on T2‐FLAIR (left) and T1‐weighted (right images). Patient 21 (slight unilateral hippocampal alteration) not illustrated. Images are neurological convention (right = right)

A total of 20 (74%) patients did not have any discernible MRI lesion. Of the seven patients with focal brain abnormalities, two had focal cortical dysplasia (7%), two had hippocampal asymmetry suggestive of hippocampal sclerosis (7%), one had multiple focal gliosis (4%), one had focal white matter hyperintensity corresponding to haemosiderin and suggestive of previous microhaemorrhages (4%), and one had focal gliosis and encephalomalacia (4%; Table 1). Figure 2 illustrates the lesional cases. Three patients (11%) had abnormalities on interictal EEG; all three patients were MRI‐negative, two experienced focal seizures with impaired awareness only, and one experienced focal to bilateral tonic–clonic seizures. Seventeen (63%) patients were seizure free after 1‐year follow‐up. Two (66%) patients with abnormal EEG and eight (33%) patients with normal EEG experienced continued seizures. Three (43%) patients with MRI‐positive findings and seven (35%) patients who were MRI‐negative experienced continued seizures.

### 3.2 Resting‐state functional MRI

**Figure 3:**
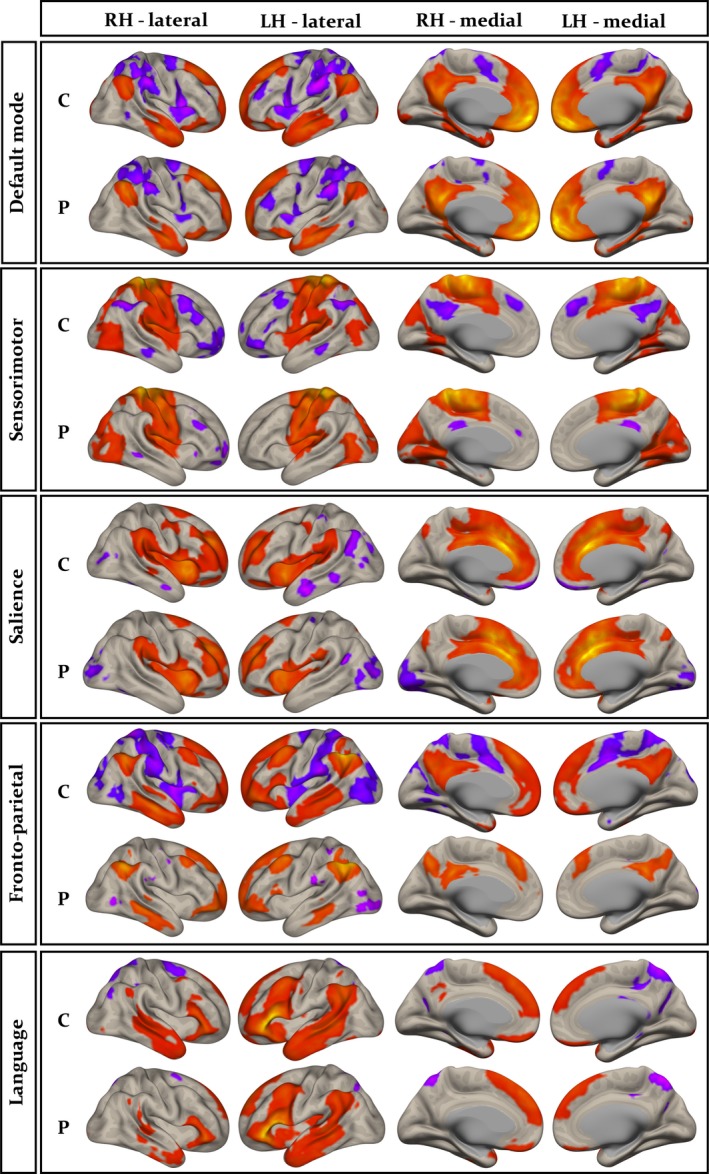
Resting‐state functional networks shown separately for controls (C) and patients (P). Regions correlated (orange) and anticorrelated (purple) with seeds are indicated. Specific seeds used to generate networks indicated here include medial prefrontal cortex (default mode), primary motor area (sensorimotor), anterior cingulate gyrus (salience), left intraparietal sulcus (fronto‐parietal), and left inferior frontal gyrus (language). Networks were reproducibly reconstructed using the alternative seeds shown in Figure 1. Note the visual difference between controls and patients in the fronto‐parietal attentional network

**Figure 4:**
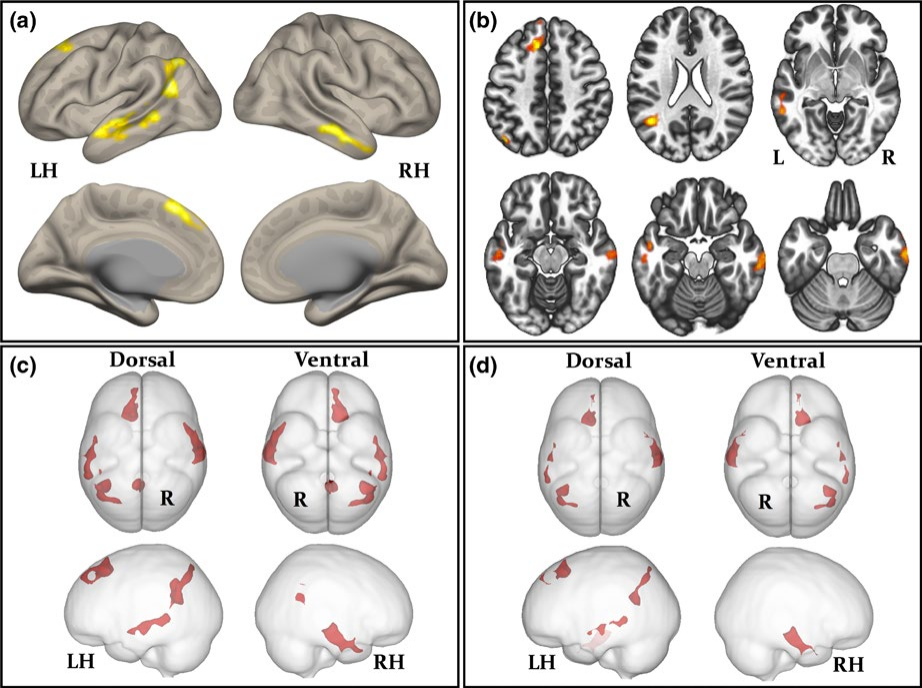
Significantly reduced functional connectivity within the fronto‐parietal attentional network in patients relative to controls (left intraparietal sulcus seed). Hypoconnectivity in all patients relative to controls is projected onto a 3D rendering (a) and axial sections (b) to illustrate anatomical locations. The spatial distribution of hypoconnectivity in all patients (c) and patients with normal MRI scans (d) is compared using glass brain projections. The corresponding information for each cluster is provided in Tables 3 and 4

Group‐wise resting‐state default mode, sensorimotor, salience, fronto‐parietal, and language networks are shown separately for controls and patients in Figure 3. The anatomical topology of each resting‐state network is indicated in Supporting Information Table S1, including the corresponding statistics, peak coordinates, and cluster size for correlated and anticorrelated voxels. Visual inspection indicated a relatively similar distribution of correlated (Figure 3, red regions) and anticorrelated (Figure 3, purple regions) networks in patients and controls for the default mode, sensorimotor, salience, and language networks. However, connectivity in the fronto‐parietal attention network was notably different between groups, manifest as a loss of connectivity within correlated and anticorrelated regions in those with epilepsy. Second‐level analyses of functional connectivity between seeds within default mode, sensorimotor, salience, and language networks and grey matter voxels across the brain revealed no significant differences between patients and controls using any seed region (Figure 1 and Table 2). However, significant differences between patients and controls were observed using the left and right intraparietal sulcus seeds within the fronto‐parietal attention network. There was significantly reduced functional connectivity between the left intraparietal sulcus seed and the right lateral temporal cortex, left lateral temporoparietal cortex, left medial frontal cortex, precuneus, and posterior cingulate cortex in patients relative to controls (Figure 4a‐c and Table 3). When analyses were performed with the seven patients with MRI‐positive findings excluded, we observed a very similar pattern of hypoconnectivity in patients relative to controls (Figure 4d and Table 4); the only difference was an absence of hypoconnectivity in the precuneus and posterior cingulate region. There was significantly reduced functional connectivity between the right intraparietal sulcus seed and right lateral temporal cortex, left mesial frontal cortex, left occipital cortex, and left cerebellum in patients relative to controls (Supporting Information Figure S1A, and Table 3). When patients with MRI‐positive findings were excluded, only significantly reduced connectivity with left mesial frontal cortex was observed (Supporting Information Figure S1B and Table 4). We found no statistically significant differences in functional networks between patients who were seizure free at follow‐up and those continued to experience seizures.

**Table 3:** Second‐level results: significantly reduced functional connectivity between intraparietal sulcus seeds and the rest of the brain in *all* patients relative to controls. Regions are illustrated in Figure 4

Seed	Anatomical regions	Peak *x*,* y*,* z*	Cluster	Cluster *p* FWE	Peak *p* unc
Left	*Right* middle temporal gyrus, temporal pole, inferior temporal gyrus	62, −12, −28	867	0.004	0.00004
	*Left* lateral occipital cortex, angular gyrus, middle temporal gyrus, supramarginal gyrus	−44, −56, 22	865	0.006	0.00001
	*Left* superior frontal gyrus, frontal pole	−10, 24, 52	672	0.007	<0.00001
	*Left* middle temporal gyrus, superior temporal gyrus, inferior temporal gyrus, temporal pole	−60, −44, −02	650	0.007	0.0002
	Precuneus, posterior cingulate gyrus	−02, −52, 20	484	0.002	0.0004
Right	*Right* lateral temporal cortex	54, −30, −16	600	0.04	0.0008
	*Left* mesial frontal cortex	−20, 40, 18	643	0.03	0.00008
	*Left* occipital cortex, cerebellum	−38, −86, −36	1,101	0.004	0.0008

**Table 4:** Second‐level results: significantly reduced functional connectivity between intraparietal sulcus seeds and the rest of the brain in *nonlesional patients only* relative to controls

Seed	Anatomical regions	Peak *x*,* y*,* z*	Cluster	Cluster *p* FWE	Peak *p* unc
Left	*Right* middle temporal gyrus, temporal pole, inferior temporal gyrus	62, −12, −28	583	0.007	0.00007
	*Left* lateral occipital cortex, angular gyrus	−44, −56, 22	570	0.009	0.00002
	*Left* superior frontal gyrus, frontal pole	−10, 24, 52	509	0.01	<0.00001
	*Left* middle temporal gyrus, superior temporal gyrus	−60, −44, −02	326	0.03	0.0001
Right	*Left* mesial frontal cortex	−20, 40, 18	451	0.05	0.0001

### 3.3 Structural MRI

There were no significant differences in grey matter or white matter structure between patients and controls using VBM at the selected statistical threshold (*p* < 0.05, FWE). There were no significant structural differences between patients who were seizure free at follow‐up and those continued to experience seizures.

## 4 DISCUSSION

There were two primary objectives of the present study. Firstly, we sought to compare resting‐state functional networks between patients and controls. We did not find connectivity alterations in patients between seeds within the default mode, sensorimotor, salience or language networks, and voxels across the brain. However, we observed significantly reduced connectivity between intraparietal seeds within the fronto‐parietal attention network and distal brain regions in patients; this hypoconnectivity was demonstrated when all patients were compared with controls and when analyses were restricted to nonlesional patients. Secondly, we sought to determine whether adults with NDfE show evidence of a common structural brain abnormality using VBM. We found no statistically significant grey matter or white matter differences between patients and controls. We discuss the biological and clinical implications of these results before highlighting pertinent methodological issues.

### 4.1 Biological and clinical implications

Our clinical data are in keeping with other reports of NDfE. In our limited sized cohort, we report that 74% of patients had a normal MRI. Other studies of NDfE in adults have reported normal MRI in 65% (Liu et al., 2002), 76% (Van Paesschen, Duncan, Stevens, & Connelly, 1997), and 78% (Van Paesschen, Duncan, Stevens, & Connelly, 1998) of patients. We reported focal cortical dysplasia in 7% and subtle signs of unilateral hippocampal sclerosis in 7% of patients. Previous reports of these abnormalities have ranged from 1.5% to 11% of adults with NDfE (Liu et al., 2002; Van Paesschen et al., 1997, 1998); 63% of our patients were seizure free after a 1‐year follow‐up, which is in keeping with large clinical studies (Annegers, Hauser, & Elveback, 1979; Kwan & Brodie, 2000; Marson et al., 2007). We found no significant association between continued seizures after AED treatment and EEG or MRI abnormality. This is likely due to the small cohort of patients with NDfE studied here in comparison with larger population studies that have reported such associations (Mohanraj & Brodie, 2013). Given that the presence of an MRI‐determined lesion is associated with medical intractability in large‐scale studies, and presumably an increasing impact of epilepsy on cognition, we may have expected that the 26% patients who were not MRI‐negative would have significantly greater alterations in network connectivity than the 74% who were MRI‐negative. However, we did not find any evidence to support this; the same functional network alterations were observed in patients when the “lesional” cases were removed from the analysis. The relative contributions of gross macroscopic lesions and impairments in functional network connectivity to cognitive impairment in NDfE need to be assessed in larger prospective studies.

To our knowledge, this is the first study of large‐scale resting‐state functional networks in patients with a new diagnosis of focal epilepsy. We report that patients with a new diagnosis of focal epilepsy have significantly reduced functional connectivity between regions within the fronto‐parietal attention network and other areas of the brain. The fronto‐parietal attentional network preferentially includes the dorsolateral and medial frontal lobe, posterior parietal cortices, and lateral temporal regions (Markett et al., 2014). Brain regions within the fronto‐parietal attention network are activated in task‐related functional MRI studies of working memory and attention (Cabeza & Nyberg, 2000; Corbetta & Shulman, 2002; Fan, McCandliss, Fossella, Flombaum, & Posner, 2005). Moreover, resting‐state functional connectivity within the fronto‐parietal network is correlated with attentional and cognitive abilities in healthy people in tasks administered outside the scanner environment (Markett et al., 2014). The significance of attentional and cognitive control processes of the fronto‐parietal network has also been demonstrated in nonhuman primates (Ptak, 2012). Furthermore, hypoconnectivity within the fronto‐parietal network has been described in other groups of patients with impaired cognitive control, such as major depressive disorder (Kaiser, Andrews‐Hanna, Wager, & Pizzagalli, 2015) and attention‐deficit/hyperactivity disorder (Lin, Tseng, Lai, Matsuo, & Gau, 2015). We therefore suggest that the loss of connectivity within this network and between this network and other regions of the brain are candidate causes of memory, executive, and attentional dysfunction that have been previously demonstrated in patients with NDfE (Aikia et al., 1995, 2001; Kalviainen et al., 1992; Prevey et al., 1998; Pulliainen et al., 2000; Taylor et al., 2010). We were, however, unable to directly address a correlation between functional brain connectivity and cognitive impairment in our sample given that our patients were not neuropsychologically evaluated. Approximately one‐half of all patients with NDfE are impaired on cognitive tasks of memory, psychomotor speed, and executive function (Taylor et al., 2010). It will therefore be interesting to determine whether it is those cognitively impaired patients who influence network hypoconnectivity, and reciprocally, whether imaging of functional networks represents a noninvasive prognostic marker of cognitive dysfunction in these patients.

We report that patient hypoconnectivity existed between intraparietal seeds and lateral temporoparietal, dorsomedial frontal, medial parietal, and occipito‐cerebellar regions. Whilst bilaterally distributed, hypoconnectivity was predominantly left lateralized regardless of whether the fronto‐parietal network was seeded from the left or right intraparietal sulcus. We cannot be certain that this lateralized effect was due to an increased number of patients with left‐sided seizure onset in our sample; a confident localization of the seizure onset zone is difficult in patients with NDfE, given that only 11% of our sample had interictal EEG abnormalities. Confident localization of the seizure focus is more likely after detailed imaging, EEG, and neuropsychological evaluation in patients with refractory focal epilepsy. The brain regions constituting the fronto‐parietal functional network are richly interconnected with white matter fibres passing through the superior longitudinal fasciculus (Ptak, 2012). It will therefore be interesting to investigate this white matter tract bundle using diffusion‐based MRI techniques in patients with NDfE.

We did not observe structural abnormalities in the group of patients relative to controls. On the one hand, this may suggest that alterations in functional networks, and concomitant effects on cognition, occur in the absence of gross focal structural abnormalities in patients with NDfE. On the other hand, whilst minimizing false positives, the stringent—but necessary—statistical approach incorporated into VBM could obscure subtle common structural alterations (Keller & Roberts, 2008). VBM has previously revealed focal alterations in groups of patients with nonlesional epilepsy who share common underlying neurobiological mechanisms (e.g., juvenile myoclonic epilepsy (O'Muircheartaigh et al., 2011; Woermann, Free, Koepp, Sisodiya, & Duncan, 1999) or temporal lobe epilepsy of unknown cause (Riederer et al., 2008; Scanlon et al., 2013)). One issue to therefore consider is that patients with NDfE have heterogeneous neurobiological mechanisms and different epileptogenic foci, which would not be identified using a technique such as VBM that is used to detect abnormalities common to a patient group. However, we suggest that there remains the possibility that common structural network alterations may exist in patients with NDfE, and which may be beyond the resolution of VBM. Particular anatomical circuits act as critical modulators of seizure generation and propagation, and seizure activity does not spread diffusely throughout the brain but propagates along specific anatomical pathways, regardless of the localization of the brain insult (Loscher & Ebert, 1996; Piredda & Gale, 1985). Furthermore, a recently published study has shown that pathological structural connectivity causes disturbances to common large‐scale functional brain networks regardless of the localization of the epileptogenic zone in patients with refractory focal epilepsy (Besson et al., 2017). Moreover, particular deep brain regions—such as the thalamus and thalamocortical pathways—play a crucial role in the clinical expression of seizures in *the epilepsies* (Dreifuss et al., 2001), and anatomically support widespread distributed cortico‐subcortical networks (Nieuwenhuys, Voogd, & Huijzen, 1988)—are structurally and physiologically abnormal in both hemispheres in patients with long‐standing focal and generalized epilepsy disorders (Bonilha et al., 2013; He, Doucet, Sperling, Sharan, & Tracy, 2015; Kay & Szaflarski, 2014; Keller et al., 2014, 2015; Kim et al., 2014; O'Muircheartaigh et al., 2012). Finally, cognitive impairment is not related to the type of focal epilepsy in those with a new diagnosis (Taylor et al., 2010). Taken together, this evidence suggests that there may be a common underlying anatomical system that is impaired in patients with NDfE. Advanced diffusion‐based MRI approaches (Bonilha et al., 2015; Glenn et al., 2016; Keller et al., 2017) may provide important insights into structural network alterations in NDfE.

### 4.2 Methodological issues

We suggest that alterations of brain functional networks may relate to cognitive dysfunction in patients with NDfE. However, we were unable to directly relate brain functional (and structural) alterations to cognitive performance in our patients, as neuropsychological assessment was not performed. This is a shortcoming of the present study. We have used our imaging data to generate the hypothesis that altered functional connectivity with seeds in the fronto‐parietal network may be related to cognitive dysfunction in NDfE by highlighting previous work that has reproducibly demonstrated (a) memory, sustained attention, executive functioning, mental flexibility, and psychomotor speed impairments in NDfE (Aikia et al., 1995, 2001; Kalviainen et al., 1992; Prevey et al., 1998; Pulliainen et al., 2000; Taylor et al., 2010) and (b) an association between the fronto‐parietal attention network and sustained attention, cognitive control, and executive functioning (Markett et al., 2014; Schmidt et al., 2016). Patients with a new diagnosis of epilepsy do not receive neuropsychological evaluation as part of their clinical assessment; such evaluation will need to be performed in the context of prospective research studies. Despite the difficulties associated with recruitment and detailed assessment of patients with NDfE—an issue that partly explains the lack of sophisticated imaging investigations in this patient group—future research should strive to simultaneously acquire neuroimaging and neuropsychological data in this understudied patient group and determine whether there is a direct link between brain functional hypoconnectivity and cognitive dysfunction.

The clinical heterogeneity and unclear seizure foci of many patients with NDfE also contribute to the lack of investigation of this patient population in neuroimaging studies. The differentiation of new‐onset focal and generalized seizures is reliably achieved through detailed assessment of seizure semiology by experienced epileptologists. However, it is difficult—and in many cases impossible—to identify the seizure focus at the time of diagnosis, which is very different to patients with long‐standing (typically refractory) focal epilepsy and well‐established foci defined using multimodal imaging and clinical investigations. The majority of patients with new‐onset seizures do not show interictal epileptiform activity on clinical EEG (Aikia, Kalviainen, Mervaala, & Riekkinen, 1999; Kim, Johnson, Marson, Chadwick, & group, M.M.S., 2006; Su et al., 2013). This is particularly true in adults, where the prognostic value of routine interictal EEG has not been established (Mohanraj & Brodie, 2013). As such, our imaging findings are “collapsed” across patients with likely newly diagnosed temporal and frontal lobe epilepsy, which constitutes the vast majority of focal epilepsies. Although recruitment of consecutive patients with NDfE naturally yields a clinically heterogeneous group, this represents a clinically pragmatic endeavour and partly accounts for the lack of sophisticated neuroimaging studies in this understudied population. We believe that there may be common markers of cognitive dysfunction and pharmacoresistance across patients with NDfE, which is supported by neuropsychological (Aikia et al., 1995, 2001; Kalviainen et al., 1992; Prevey et al., 1998; Pulliainen et al., 2000; Taylor et al., 2010) and imaging (Kim, Kim, Lee, & Park, 2017) work. The study by Kim et al. (2017) reported that patients with NDfE who continued to experience seizures despite AED therapy had reduced volumes of the corpus callosum relative to healthy controls and patients who were rendered seizure free. The identification of a common biomarker for cognitive and treatment outcome in patients with NDfE represents an important future research endeavour.

## 5 CONCLUSION

We have demonstrated that patients with NDfE have significantly reduced connectivity between seeds within the fronto‐parietal attention functional network and other cortical regions. This loss of connectivity is not influenced by the presence of a gross macroscopic epileptogenic lesion. This work indicates that functional brain abnormalities are not necessarily a consequence of the chronicity of epilepsy and are present when seizures first emerge.

## CONFLICT OF INTEREST

The authors declare no competing financial interests.
