# Supplementary material for: Resting‐state functional brain networks in adults with a new diagnosis of focal epilepsy
Source: Brain Behav. 2018 Nov 28;9(1):e01168. doi: 10.1002/brb3.1168 (PMC6346674; doi:10.1002/brb3.1168)
Supplement: Supplementary file 1 [file BRB3-9-e01168-s001.docx]

**Resting-state functional brain networks in adults with**

**a new diagnosis of focal epilepsy**

**Figure S1**


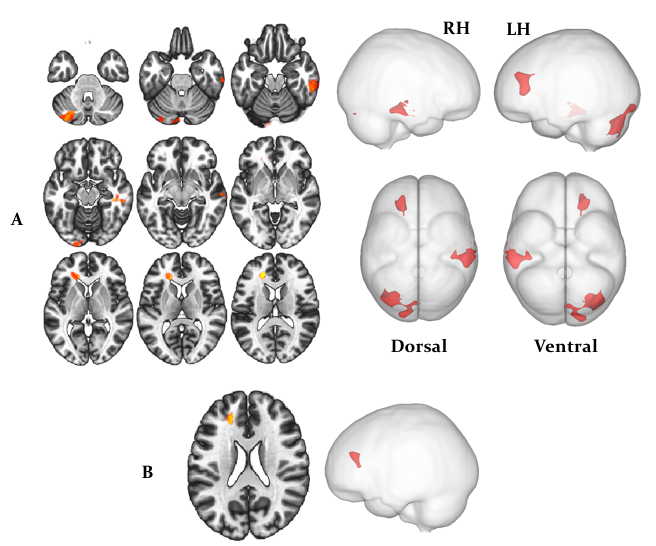


Significantly reduced functional connectivity with the fronto-parietal attentional network in patients relative to controls (right intraparietal sulcus seed). A. Hypoconnectivity in all patients relative to controls are projected onto axial sections (left) and glass brain projections (right). B. Significant hypoconnectivity with the same network only in patients with normal MRI scans. The corresponding information for each cluster is provided in Tables 3 and 4 in the main article.
